# Physical activity benefits of attending a senior center depend largely on age and gender: a study using GPS and accelerometry data

**DOI:** 10.1186/s12877-020-01527-6

**Published:** 2020-04-15

**Authors:** Oriol Marquet, Monika Maciejewska, Xavier Delclòs-Alió, Guillem Vich, Jasper Schipperijn, Carme Miralles-Guasch

**Affiliations:** 1 IS Global (Barcelona Institute for Global Health), Barcelona, Spain; 2 Geography Department, Autonomous University of Barcelona, Barcelona, Spain; 3 Research Unit for Active Living, Department of Sports Science and Clinical Biomechanics, University of Southern Denmark, Odense, Denmark

**Keywords:** Senior center, Accelerometry, Physical activity, MVPA, GPS tracking

## Abstract

**Background:**

Senior centers offer important opportunities for physical activity and social interaction. Seniors who visit a senior center regularly can gain physical activity from transportation and from specific activities offered within the senior center. However, there is very little knowledge regarding the specific physical activity gains obtained from regular visits to senior centers, and no effort has been made to use device-based measures of physical activity to test the potential physical activity benefits of attending a senior center.

**Methods:**

To fill this gap, the present study examined the physical activity patterns of 227 seniors living in the Barcelona Metropolitan Area in Spain. Using GPS and Accelerometer 7-day tracking data, and GIS measures we assessed the light physical activity and moderate-to-vigorous intensity physical activity (MVPA) benefits of attending the senior center on a weekly and daily basis.

**Results:**

Seniors who attended a senior center at least once a week did not accumulate significantly more daily physical activity (211.6 min; 95% CI 196.6; 226.6) than seniors without any visit 215.9 min; 95% CI 202.7; 229). However, on a day-to-day basis, it was found that visiting a senior center had positive effects in physical activity and was associated with less sedentary time among younger participants in general (− 18.2 daily min 95% CI − 33.2;-3.3 ***p*** = 0.016) and among older female participants in particular (− 19.7 daily min 95% CI -21.06;-18.5 ***p*** = 0.011).

**Conclusions:**

The benefits of attending senior centers in terms of physical activity should not be viewed as universal, but rather as contingent to the demographics of the user, and the type of activity that the visit is replacing.

## Background

Physical activity is a key part of active aging and the association between exercise and physical health is well established. Exercise at advanced ages is important to maintain physical fitness, and it can promote mobility, prevent falls and also provide access to opportunities that help personal independence [1]. Physical activity (PA) is considered a key part of maintaining quality of life and successful aging [2] as there is abundant evidence that individuals who engage in higher levels of PA tend to have higher health-related quality of life (HRQoL) scores [3], and better physical health [4–6].

Senior centers, also commonly known as elderly centers or seniors’ clubs, offer a wide variety of programs and services. By offering opportunities for social interaction and friendship, senior centers have traditionally had a central role in easing loneliness, increasing social integration and reducing isolation [7–9]. In the case of senior centers in Spain, these public facilities offer a range of free activities provided by health care professionals that can range from knitting classes, formal or informal board games such as playing cards, dominoes or chess, to organized physical activity classes such as yoga, Zumba or aerobics. These physical activity classes are adapted to seniors and tailored to improve and maintain their physical capacity while also providing fun and attractive environments. In recent years, with the rise in popularity of concepts such as active aging and aging in place, the role of senior centers as providers and promoters of physical activity has gained relevance. In the context of walkable and accessible urban environments [10, 11], senior centers can act as mediators of active and successful aging [12]. However, there is an important lack of research devoted to measuring and objectively assessing the physical activity that seniors gain from their visits to senior centers.

Previous research on senior centers and physical activity has focused on evaluating how the change in a specific program available in senior centers can increase the participants’ physical activity [2, 13–16]. However, as demonstrated by Felix et al., [17] not all senior centers can program these specific activities and there is a need to explore how attending a regular senior center can affect physical activity. Schmitt et al. [18] are the only researchers who studied seniors in daycare centers outside programmed activities and they found no significant differences on physical activity when comparing attendees and non-attendees. Further, Kim et al., [19] found attendees to senior centers to score higher in physical functionality but those authors did not actually measure physical activity.

Other potential benefits of senior centers such as socialization and reducing isolation have drawn much more attention. Studies in the body of literature have found positive associations between senior center attendance and self-confidence in seniors’ own abilities [20], and having an increased number of acquaintances and friends [21, 22]. Consistently, attending day programs at senior centers has also been found to delay institutional and nursing home placement [23, 24] as well as improving wellbeing and mental wellness and reducing depression rates [19]. Ron [25] found that regular attendance to senior centers contributed to the self-esteem of older females, while Iecovich and Biderman [26] found that users of adult day care centers had significant quality of life benefits but found no evidence that users of senior centers used less health services compared with nonusers [27].

Similarly, a large amount of literature has been devoted to the determinants of senior center attendance [28, 29] and some studies have reported female seniors to use these centers more often than male seniors [7, 30], while others have found no significant relationship between gender and senior center attendance [31, 32].

As demonstrated by two recent reviews, the existing literature has mainly focused on what happens inside the senior center but there is a lack of information about seniors’ lives beyond the center [33] and more research on the individual effects of senior center attendance is needed [34]. With the notable exception of Turner et al. [35], who used pedometers to measure seniors’ weekly physical activity, to our knowledge no other study has used a device-based measurement of physical activity. Similarly, to date no study has attempted to analyze how senior center attendance is changing the physical activity of seniors with no specific participation in programs or activities.

To fill the prominent research gaps noted in the above, we aimed at measuring the physical activity benefits of visiting a senior center using GPS and Accelerometer 7-day tracking data for 227 seniors in the Metropolitan Region of Barcelona, Spain. Our goal was to measure 1) the weekly physical activity of seniors who use the services of the senior center and compare it with those who did not use the services, and 2) measure the daily physical activity of users and compare the physical activity of those users who attended the senior center on that particular day, with those users who did not attend.

## Methods

### Recruitment process

For this study we used a convenience sample of 227 seniors (over 65 years of age) living in the Barcelona Metropolitan Region, Spain. Data were collected in the period of 2017–2018 and analyzed in 2019. We contacted 39 public and private senior centers in search of potential participants. After an initial screening of the interested senior centers, a pair of researchers traveled to each senior center that had provided positive feedback (*n* = 20) and explained the research protocol to all the seniors who had expressed an interest in this study. In those sessions the functioning and maintenance of the devices were explained, together with the nature of the data collected. All willing participants were asked to sign a consent form before they were given an accelerometer and GPS device and asked to answer a first short survey regarding their sociodemographic characteristics. Additionally, a snowball (chain-referral) technique, which asks participants to seek volunteers in their social circle, was used to contact other seniors that lived in the area but did not attend the senior centers. As an incentive, all participants received a report on their own physical activity patterns at the end of the study. This report summarized their physical activity during participation and provided advice on how to increase their physical activity in the future.

### Study design

Participants were asked to wear a GPS device and an accelerometer for 7 days. Additionally, all participants answered a questionnaire about their sociodemographic characteristics, daily mobility and physical activity patterns and perceived health status. Body Mass Index (BMI) was calculated based on self-reported height and weight. The study received the approval of the Autonomous University of Barcelona (UAB is the Spanish acronym) institutional review board (CEEAH-3656).

#### Accelerometer data processing

Data from 7-day accelerometer tracking provided estimates of energy expenditure. Participants had to wear ActiGraph accelerometers (model GT3X+) for a minimum of 10 waking hours per day and the gathered data were included in the analysis. Following Kamada et al., [36] a threshold of < 2000 VM counts per min was used to define sedentary time, > 2000 for light, and > 7500 and > 8500 for moderate to vigorous physical activity (MVPA), respectively. GPS data were collected using the QStarz BT-Q1000X device at intervals of 15 s. Accelerometer and GPS data were merged and filtered using PALMS software [37] and further categorized into minutes spent being sedentary, in light and moderate-to-vigorous physical activity (MVPA) per day.

### Sample

The main demographic characteristics of both senior center users and non-users were highly similar (Table 1). Senior center users were slightly older and reported slightly higher weekly physical activity. Both groups had no differences in access to a senior center, as represented by their estimated walking time to the nearest senior center. The sample was also balanced in terms of gender with women being slightly overrepresented in both groups.

**Table 1:** Characteristics of the sample

	Senior center members	Non-members	***P***^**a**^
***Sample***			
N (individuals)	97	130	
N of valid days	654	848	
Days per participant (Avg)	6.74	6.52	
***Demographics***			
Average Age (SD)	76.6 (5.9)	73.5 (7.3)	
Gender			0.966
Male	43 (44.3%)	58 (44.6%)	
Female	54 (55.7%)	72 (55.4%)	
***Health***			
BMI			0.726
Normal weight	18 (18.6%)	22 (16.9%)	
Overweight + obesity	65 (67%)	70 (53.8%)	
nd.	14 (14.4%)	38 (29.2%)	
Self-reported Health			0.262
Poor	4 (4.1%)	3 (2.3%)	
Regular	28 (28.9%)	40 (30.8%)	
Good	65 (67%)	98 (52.3%)	
Weekly PA minutes (SD)^b^	285.9 (317.8)	225.3 (234.3)	0.235
***Mobility***			
Usual mode of transport			0.420
Walking	57 (58.7%)	89 (68.5%)	
Public	18 (18.6%)	19 (14.6%)	
Private	20 (20.6%)	22 (16.9%)	
Travel time to senior center^c^			0.558
1–10 min	60 (61.9%)	71 (54.6%)	
11–20 min	20 (20.6%)	33 (25.4%)	
> 20 min	17 (17.5%)	19 (14.6%)	
nd.	0 (0%)	7 (5.4%)	

^a^ Based on chi-Square statistics for categorical variables and ANOVA for continuous variables

^b^ Self-reported minutes of physical activity obtained using the IPAQ short questionnaire

^c^ Self-reported travel time (in minutes) that it would take to walk from home to the closest senior center

### Measures

Visits to senior center: To determine whether a participant had visited a senior center during any of their 7 participation days, we used a 25 m circular buffer around the senior center. All participants with 20 or more indoor minutes spent within that buffer were considered to have visited the senior center during that day. The interior of the 25 m buffers were screened in search of grocery stores, gyms, medical centers, libraries or other facilities that could gather seniors for substantial periods of time. Participants who lived within 100 m of a senior center (*n* = 21) were excluded from the analysis due to the impossibility of clearly determining the extent of their visits to a senior center.

Physical activity: We used the total minutes of sedentary time, light physical activity (LPA) and moderate-to-vigorous activity (MVPA) of each participant for each participated day as main measures of physical activity. We added the total minutes of light, moderate and vigorous activity to calculate the total minutes of activity recorded during the day by each participant. This included physical activity gained while at home, while traveling and also while at the senior center.

### Analysis

To examine associations between attending the senior center and the total daily amount of physical activity, we used descriptive statistics followed by multilevel linear mixed effects models with user ID as a random effect. First, we used one-way analysis of variance (ANOVA) to test the differences in weekly physical activity behaviors, expressed as total sedentary time, LPA and MVPA, between those participants who attended the senior center at least one day out of the 7 total participation days, and those who had not attended the senior center. We then focused only on senior center users (*n* = 97), and tested the day-to-day difference in PA between the days on which they attended the senior center, and the days on which they did not attend.

Finally, we regressed the attendance to a senior center per day onto each of the PA levels, sedentary, LPA, and MVPA, using separate multilevel mixed models. These models included having visited a senior center (1 = yes; 0 = no) as the dependent variable and gender (M; F), age (< 75 yrs.; ≥75 yrs), total device wear-time, distance to the closest senior center (self-assessed; < 10 min, 10–20 min, 20+ min), perceived health (self-assessed; poor, regular, good), and presence of rain (1 = Rain; 0 = None) as main control variables. The control for the presence of rain is due to some studies having found that weather conditions significantly alter the travel behavior and activity participation of older people [38] due to the weather conditions impacting on the decisions of older people regarding leaving their homes.

To better understand the importance of attending the senior center for each gender and age group combination, we created three-way interaction terms for the variables of age, gender, and senior center attendance and then calculated the daily physical activity-adjusted predictions at each representative value using the Stata 15 “margins” command. These actions resulted in the estimated number of minutes of each PA type per category of age, gender, and senior center attendance. We then used the Stata 15 “contrast at sample means” post-estimation command to test the significance of the estimation differences for between-groups.

## Results

The final sample comprised of 227 individuals who accumulated 1502 valid days of data. From this sample, 97 individuals were identified as regular senior center users, while 130 individuals where non-users. From an initial sample of 269 seniors contacted, only 227 were included in the analysis due to ineligibility (i.e., they did not leave their residence at least once a day or they presented signs of dementia) or the fact of their insufficient participation in the study.

Seniors who attended a senior center at least once during the participation week accumulated more weekly sedentary time than seniors who had not visited a senior center at all (6789 min vs. 6307 min), although the differences at the aggregated level were not significant (Table 2). As expected, older seniors were significantly less active than younger seniors, while no major gender differences were found. Visiting a senior center was not associated with any level of PA across any gender and age group, with the exception of Male senior center users, who were more sedentary than their male non-senior center user counterparts (6302 min vs. 7140 min; *p* = 0.03).

**Table 2:** Physical activity patterns of participants that attended a senior center at least once during the week, and senior participants that did not

	N^e^	Sedentary^a^			LPA^b^			MVPA^c^			Total PA^d^		
		mean	SD	*p*^f^	mean	SD	*p*^f^	mean	SD	*p*^f^	mean	SD	*p*^f^
Senior center				0.054			0.899			0.695			0.917
Non-user	130	6307	1963		1405	538		17.5	30.3		1422	550	
User	97	6789	1705		1414	527		16.1	22.7		1430	538	
Gender				0.295			0.118			0.592			0.133
Female	126	6396	1822		1458	555		16	18.8		1474	563	
Male	101	6658	1924		1347	499		17.9	35.2		1364	515	
Age				0.278			0.001*			0.021*			0.001*
< 75	109	6373	1839		1531	472		21.2	26.4		1552	483	
≥75	118	6642	1894		1296	562		12.8	27.6		1301	573	
Female				0.546			0.566			0.270			0.547
Female, non-user	72	6311	1890		1433	565		14.4	14.6		1448	571	
Female, user	54	6510	1738		1491	546		18.1	23.3		1509	557	
Male				0.03*			0.605			0.266			0.564
Male, non-user	58	6302	2065		1369	507		21.3	42.3		1391	527	
Male, user	43	7140	1616		1317	492		13.4	13.4		1330	503	
< 75				0.394			0.889			0.806			0.881
< 75, non-user	75	6271	1878		1526	442		20.8	28.2		1547	453	
< 75, user	34	6597	1753		1540	540		22.6	22.2		1562	550	
≥75				0.124			0.305			0.965			0.315
≥75, non-user	55	6355	2087		1239	614		13	32.8		1252	625	
≥75, user	63	6893	1684		1346	512		12.7	22.5		1359	523	
Total	227	6512	1869		211.5	73.2		2.5	4.1		214.1	74.9	

^a^ Daily time spent sedentary (minutes)

^b^ Daily time spent in LPA (minutes)

^c^ Daily time spent in MVPA (minutes)

^d^ Daily time spent in physical activity (light, moderate or vigorous) (minutes)

^e^ Number of participants

^f^ One way ANOVA

* Statistically significant value

When focusing only on the bivariate association of those participants who had attended the senior center at least once and their daily physical activity (Table 3), we can see how attending the senior center was not associated with higher daily levels of physical activity, but it was associated with less sedentary time. On a daily basis, female senior center attendees accumulated more light physical activity time than men (222.2 min vs. 190.2 min; *p* = 0.024) and more total physical activity (224.9 vs. 192.1 min; *p* = 0.023).

**Table 3:** Senior center users only, comparison between days on which they use it, and days on which they do not

	Sedentary^a^			Light^b^			MVPA^c^			Total PA^d^		
	mean	SD	*p*^e^	mean	SD	*p*^e^	mean	SD	*p*^e^	mean	SD	*p*^e^
Senior center			0.01*			0.622			0.213			0.537
Attended	1007.9	21.6		206.9	7.5		2.1	0.4		209.0	7.7	
Didn’t attend	969.7	22.8		209.6	7.9		2.6	0.4		212.4	8.1	
Gender			0.076			0.024*			0.237			0.023*
Female	960.7	27.6		222.2	9.5		2.7	0.4		224.9	9.7	
Male	1014.8	30.8		190.2	10.5		1.9	0.5		192.1	10.8	
Age			0.053			0.041			0.032			0.036*
< 75	978.6	34.9		227.3	11.8		3.2	0.5		230.6	12.1	
≥75	1001.6	26.0		197.2	8.8		1.8	0.4		199.0	9.0	
Female			0.001*			0.746			0.001*			0.596
Attended	989.4	27.9		221.4	10.1		2.0	0.5		223.3	10.3	
Didn’t attend	907.6	29.5		223.9	10.9		3.8	0.6		227.8	11.1	
Male			0.54			0.651			0.106			0.733
Attended	1028.2	32.1		188.8	10.6		2.3	0.5		191.0	10.9	
Didn’t attend	1042.9	33.6		192.1	11.1		1.3	0.6		193.1	11.3	
< 75			0.333			0.224			0.16			0.183
Attended	987.9	34.3		222.9	12.3		2.8	0.6		225.6	12.6	
Didn’t attend	962.9	36.9		235.2	13.4		4.0	0.7		239.3	13.7	
≥75			0.011*			0.659			0.745			0.705
Attended	1019.3	27.6		198.3	9.2		1.8	0.4		199.9	9.4	
Didn’t attend	973.5	28.9		195.5	9.7		1.9	0.5		197.5	9.9	

^a^ Daily time spent sedentary (minutes)

^b^ Daily time spent in LPA (minutes)

^c^ Daily time spent in MVPA (minutes)

^d^ Daily time spent in physical activity (light, moderate or vigorous) (minutes)

^e^ One way ANOVA

* Statistically significant value

Among the females, having attended the senior center was associated with more daily sedentary time (989.6 min vs. 907.6; *p* = 0.001) and with less MVPA (2.0 min vs. 3.8; *p* = 0.001). Similarly, on this aggregated level having attended a senior center during the day was also found to be associated with more sedentary time among older people (75 yrs. of age +) (1019.3 min vs. 973.6; *p* = 0.011).

It is noteworthy that the results changed when the relationship between senior center attendance and physical activity is assessed while taking into account the interactions of age and gender, together with several control variables. Post-estimating the adjusted predictions at each representative value of age and gender after using a multilevel mixed model (see Supplementary Table 1) we can obtain the estimated physical activity of senior center attendees for each age and gender group while adjusting for several covariates (Table 4).

**Table 4:** Adjusted^a^ estimated minutes of physical activity per age and gender on days with a senior center visit, and days without a visit

	Sedentary^b^				LPA^c^				MVPA^d^			
	SC visit^e^	No visit^f^	Diff	%Diff	SC visit	No visit	Diff	%Diff	SC visit	No visit	Diff	%Diff
Female												
< 75	962.1	977.4	−15.2	−1.6%	248.1	235.7	12.4	5.0%	5.9	3.2	2.7*	46.0%
≥75	985.2	1005.0	−19.8^*^	−2.0%	228.1	210.0	18.0^*^	7.9%	2.8	1.3	1.5*	52.6%
Male												
< 75	1002.5	1024.4	−21.9	−2.2%	212.3	190.0	22.3	10.5%	1.4	1.9	−0.4	−30.5%
≥75	1033.1	1021.5	11.6	1.1%	181.7	192.3	−10.6	−5.8%	1.3	2.6	−1.3	− 102.6%
All												
< 75	980.2	998.5	−18.2^*^	1.8%	232.0	215.2	16.8^*^	7.3%	3.9	2.6	1.3	33.4%
≥75	1006.7	1012.4	−5.7	0.6%	207.2	202.1	5.2	2.5%	2.1	1.9	0.2	10.5%

^a^Adjusted by total device wear time, distance to the closest senior center, perceived health, and presence of rain

^b^ Daily time spent sedentary (minutes)

^c^ Daily time spent in LPA (minutes)

^d^ Daily time spent in MVPA (minutes)

^e^ Values for days on which participants have visited a senior center

^f^ Values for days on which participants have not visited a senior center

^*^ Contrast of estimates between groups (senior center vs no senior center) found significant (*p* < 0.05)

It was estimated that females younger than 75 yrs. of age accumulated 2.7 more minutes of MVPA on the days when they had attended the senior center (*p* = 0.002). This represented a 46% increase from the MVPA gained on the days when they had not attended the senior center. Among older women (> 75 yrs. of age), attending the senior center was associated with an estimated 19.8 less sedentary minutes per day (*p* = 0.001), 18 min more LPA (*p* = 0.018) and 1.5 min more MVPA (*p* = 0.032). By contrast, males attendance at the senior center was not associated with any physical activity level, regardless of age. Despite that, it is still noteworthy how for older men (> 75 yrs. of age) visiting the senior center seems to have negative consequences because the visits are associated with more sedentary time, and less light and moderate to vigorous physical activity.

Overall, the differences in trends between men and women of older age (> 75 yrs. of age) present on an aggregated level no significant associations between attending the senior center and any domain of physical activity. However, because the associations of both male and female younger seniors (< 75 yrs. of age) were in the same direction, they reached statistical significance when observed on the aggregated level. Attending the senior center was associated with an estimated 18 min less sedentary time (*p* = 0.017) and 16.8 min more LPA (*p* = 0.025).

## Discussion

This paper has explored the physical activity benefits that attending a senior center can have for seniors using the objective 7-day physical activity tracking of 227 seniors living in the Metropolitan Region of Barcelona, Spain. Physical activity data were obtained using accelerometers, while attendance to senior centers was obtained using GPS measures and GIS processing. Results suggest that the benefits of attending a senior center are not universal and depend largely on age and gender.

Our results show that older seniors (≥75 yrs. of age) in our study engaged in significantly less weekly LPA and MVPA than their younger counterparts but no statistical differences between men and women were found, which is consistent with the previous studies in the body of literature [35, 39]. Seniors who attended a senior center at least once did not accumulate more weekly physical activity than seniors with no visit. These results concur with the findings by Turner et al. [35] who failed to find a significant association between attending a senior center physical activity program and increased physical activity. In a similar study, Iecovich and Biderman [26] found that attending a senior center produced a number of benefits in terms of quality of life, but did not include physical activity.

This would suggest that we should not automatically assume that visiting a senior center has positive benefits in terms of physical activity. Our results however might be affected by demographic differences between senior center users and non-users. Non-users were slightly older and reported slightly better health status than those attending the senior center, which is consistent with age and health being previously identified as the major determinant of senior center attendance [7, 30, 40].

As demonstrated by Whaley and Ebbeck [41], active seniors can be reluctant to go to the senior center, because it is a place that they associate with sedentary activity, older people or people with difficulties [30]. At the individual level the benefits of attending a senior center can only be understood in relation to the activity that this visit is replacing. If a senior visits the senior center instead of staying at home engaging in a sedentary activity, then the senior center will have helped to increase the physical activity of that senior on that day. Conversely, if the visit to the senior center replaces a trip to the supermarket or an outdoor yoga class, this visit will have probably contributed negatively to the physical activity balance at the end of the day. As a result, attendance at senior centers cannot be assessed in a vacuum, but rather in the context of the physical activity patterns of each population group in terms of age and gender. For more active groups, senior centers will probably be less beneficial than for traditionally more sedentary groups.

Therefore, it is also important to measure daily physical activity rates while adjusting for several important covariates that might be affecting the overall amount of physical activity. In this study, after adjusting for total device wear-time, distance to the senior center, perceived health, and presence of rain, visiting a senior center was found to be associated with less sedentary time and more light physical activity among seniors younger than 75 yrs. of age. These findings extend those of Fawcett et al. [22] who note that seniors in their study below 70 yrs. of age gained greater benefits from visiting the senior center in terms of wellbeing, confidence, empowerment and socializing.

However, following gender stratification of the population sample, we found that the group of female participants gained greater benefits from attending a senior center. The female participants increased their light physical activity by 8%, their MVPA by 52.6% and they decreased their sedentary time by 2%. For this group, having a structured activity during the day might encourage them to leave home and contribute to engaging in some additional activities besides going to the senior center.

For the older male seniors (> 75 years of age) in contrast, going to the senior center seemed to have mainly negative consequences, although the findings were not statistically significant. Those older male seniors who attended the senior center were found to be more sedentary and less active than those older male seniors who had not attended the senior center during the day, although the differences were not found to be significant. Older male adults in general were in fact the only demographic group for which going to a senior center had negative consequences in terms of physical activity. These results confirm the findings of Swan et al., [42] who observed in their study that senior males were less likely than females to participate in senior center physical activity sessions. This can be explained either by a preference of older male adults to engage in sedentary activities while at the senior center, such as card playing or newspaper reading, or by their usual activities outside the senior center being more particularly active, such as strolling. These results suggest that in the case of older men, activities and programs in senior centers should be focused towards increasing their physical activity and discouraging the more sedentary activities.

Overall, these findings should inform the program directors in senior centers to focus on providing more exercise-related activities at the same time as aiming to avoid more sedentary activities. The goal however needs to be an increase in the physical activity of seniors without losing other benefits of senior centers such as socializing, developing confidence and exercising cognitive abilities. Thus, these more lively activities should also incorporate cognitive tasks, in order not to lose the psycho-social benefits of typical senior center activities [43].

To the best of our knowledge, this paper has been the first to use GPS and senior center attendance to assess the physical activity benefits of attending a senior center during the day. We have used accelerometer data to go beyond self-assessment of physical activity and GPS data to detect senior center attendance. Based on these findings, our recommendation is that future programming should aim to increase the physical activity of both senior men and women in the age range of 65–75 yrs. in order to add some form of physical activity to the long list of benefits that senior centers have for the wellbeing and quality of life of the elderly within the society.

This study is not without limitations. First, the sample might be biased towards people whose general health conditions are good enough for them to be willing to participate in the study, hence they actually might currently be more active than the ‘average’ type of senior citizen. Similarly, the location of the senior centers was chosen randomly and did not follow a pre-established scheme. The GPS process used to detect senior center visits was based only on indoor time spent in the proximity of a senior center. Despite the efforts made on screening other confounding places that could also gather seniors, that still leaves some room for error in mislocating some physical activity with respect to a senior center, when in fact the physical activity was made outside of the senior center. Unfortunately, in this study, some relevant variables regarding socioeconomic and demographic characteristics such as marital status, income or education were not available to us. Finally, no information was available on the type of activity available at the time of the senior center visit by the participating seniors. It was thus not possible to control for the presence of organized activities, that could be both attracting seniors to the senior center and motivating those senior citizens to lead a more active lifestyle.

## Conclusions

According to the data in this study, the benefits of visiting senior centers were not universal regarding the study participants, but they were rather contingent on socioeconomic groups and alternative behaviors. Visiting a senior center was found to have a positive effect particularly among older females, for whom the senior center doubled the total daily amount of recorded MVPA. In contrast, older males were found to have a negative association between visiting a senior center and their daily physical activity. A further evaluation of the causes behind these differences is necessary, one that takes into account what kind of behavior and types of activities that the visit to a senior center would be replacing. However, the results of the present study demonstrate the need to promote more intensive senior center activities among the older male population at the same time as validating encouraging senior center attendance as a healthy policy for the older female population.

## Supplementary information

**Additional file 1: Table S1** Multilevel linear regressions per type of daily physical activity and participant characteristics.

## Data Availability

The datasets used and analyzed during the current study are available from the corresponding author on any reasonable request.

## Abbreviations

PA: Physical activity
LPA: Light physical activity
MVPA: Moderate-to-vigorous physical activity

## References

[1] Dattilo J, Lorek AE, Mogle J, Sliwinski M, Freed S, Frysinger M (2015). Perceptions of leisure by older adults who attend senior centers. Leis Sci.

[2] Hand BD, Cavanaugh S, Forbes W, Govern J (2012). Changes in health-related quality of life and functional fitness with exercise training in older adults who attend senior centers. Act Adapt Aging.

[3] Shibata A, Oka K, Nakamura Y, Muraoka I (2007). Recommended level of physical activity and health-related quality of life among Japanese adults. Health Qual Life Outcomes.

[4] Tomas-Carus P, Biehl-Printes C, Raimundo A, Laranjo L, Pereira C, Terra NL (2014). A cross-sectional study on physical and sedentary activity and health-related quality of life in institutionalized vs . non-institutionalized elderly Um estudo transversal sobre atividade física e qualidade de vida relacionada. Pan Am J Aging Res.

[5] Buchman AS, Wilson RS, Yu L, James BD, Boyle PA, Bennett DA (2014). Total daily activity declines more rapidly with increasing age in older adults. Arch Gerontol Geriatr.

[6] Hipp JA, Dodson EA, Lee JA, Marx CM, Yang L, Tabak RG (2017). Mixed methods analysis of eighteen worksite policies, programs, and environments for physical activity. Int J Behav Nutr Phys Act.

[7] Bøen H, Dalgard OS, Johansen R, Nord E (2010). Socio-demographic, psychosocial and health characteristics of Norwegian senior Centre users: a cross-sectional study. Scand J Public Health.

[8] Rosenberg BC (2015). Social spaces for seniors: exploring seniors’ centres and clubs in Australia. J Sociol.

[9] Boen H, Dalgard OS, Johansen R, Nord E. A randomized controlled trial of a senior Centre group programme for increasing social support and preventing depression in elderly people living at home in Norway. BMC Geriatr. 2012;12(1):20. 10.1186/1471-2318-12-20 PMC3494554 22607553

[10] Marquet O, Miralles-Guasch C (2015). Neighbourhood vitality and physical activity among the elderly: the role of walkable environments on active ageing in Barcelona, Spain. Soc Sci Med.

[11] Marquet O, Hipp JA, Miralles-Guasch C (2017). Neighborhood walkability and active ageing: a difference in differences assessment of active transportation over ten years. J Transp Heal.

[12] Marhánková JH (2014). “Women are just more active” - gender as a determining factor in involvement in senior centres. Ageing Soc.

[13] Wallace JI, Buchner DM, Grothaus L, Leveille SG, Tyll L, LaCroix A (1998). Implementation and effectiveness of a community-based health promotion program for older adults. Journals Gerontol - Ser A Biol Sci Med Sci.

[14] Sarkisian CA, Prohaska TR, Davis C, Weiner B (2007). Pilot test of an attribution retraining intervention to raise walking levels in sedentary older adults. J Am Geriatr Soc.

[15] Moore-Harrison TL, Johnson MA, Quinn ME, Cress ME (2016). An evidence-based exercise program implemented in congregate-meal sites. J Phys Act Health.

[16] Porter KN, Fischer JG, Johnson MA (2011). Improved physical function and physical activity in older adults following a community-based intervention : relationships with a history of depression. Maturitas..

[17] Felix HC, Adams B, Cornell CE, Fausett JK, Krukowski RA, Love SRJ (2014). Barriers and facilitators to senior centers participating in translational research. Res Aging.

[18] Schmitt EM, Sands LP, Weiss S, Dowling G, Covinsky K (2010). Adult day health center participation and health-related quality of life. Gerontologist..

[19] Kim HS, Harada K, Miyashita M, Lee EA, Park JK, Nakamura Y (2011). Use of senior center and the health-related quality of life in Korean older adults. J Prev Med Public Heal.

[20] Dabelko-Schoeny H, King S (2010). In their own words: participants’ perceptions of the impact of adult day services. J Gerontol Soc Work.

[21] Aday RH, Kehoe GC, Farney LA (2006). Impact of senior center friendships on aging women who live alone. J Women Aging.

[22] Fawcett B (2014). Well-being and older people: the place of day clubs in reconceptualising participation and challenging deficit. Br J Soc Work.

[23] Kelly R, Puurveen G, Gill R (2016). The effect of adult day services on delay to institutional placement. J Appl Gerontol.

[24] Gaugler JE, Kane RL, Kane RA, Newcomer R (2005). Early community-based service utilization and its effects on institutionalization in dementia caregiving. Gerontologist..

[25] Ron P (2007). Self-esteem among elderly people receiving care insurance at home and at day centers for the elderly. Int Psychogeriatrics.

[26] Iecovich E, Biderman A (2013). Quality of life among disabled older adults without cognitive impairment and its relation to attendance in day care centres. Ageing Soc.

[27] Iecovich E, Biderman A (2013). Use of adult day care centers: do they offset utilization of health care services?. Gerontologist..

[28] Iecovich E, Biderman A (2012). Attendance in adult day care centers of cognitively intact older persons: reasons for use and nonuse. J Appl Gerontol.

[29] Iecovich E, Carmel S (2011). Differences between users and nonusers of day care centers among frail older persons in Israel. J Appl Gerontol.

[30] Pardasani M (2010). Senior centers: characteristics of participants and nonparticipants. Act Adapt Aging.

[31] Lai DWL (2008). Predictors of use of senior centers by elderly Chinese immigrants in Canada. J Ethn Cult Divers Soc Work.

[32] Walker J, Bisbee C, Porter R, Flanders J (2004). Increasing practitioners’ knowledge of participation among elderly adults in senior center activities. Educ Gerontol.

[33] Orellana K, Manthorpe J, Tinker A (2019). Day centres for older people: a systematically conducted scoping review of literature about their benefits, purposes and how they are perceived. Ageing Soc.

[34] Kadowaki L, Mahmood A (2018). Senior Centres in Canada and the United States: a scoping review. Can J Aging.

[35] Turner MJ, Schmitt EE, Hubbard-Turner T (2016). Weekly physical activity levels of older adults regularly using a fitness facility. J Aging Res.

[36] Kamada M, Shiroma EJ, Harris TB, Lee IM (2016). Comparison of physical activity assessed using hip- and wrist-worn accelerometers. Gait Posture.

[37] Jankowska MM, Schipperjin J, Kerr J (2015). A framework for using GPS data in physical activity and sedentary behavior studies. Exerc Sport Sci Rev.

[38] Mitra R, Siva H, Kehler M (2015). Walk-friendly suburbs for older adults? Exploring the enablers and barriers to walking in a large suburban municipality in Canada. J Aging Stud.

[39] Strath SJ, Swartz AM, Cashin SE (2009). Ambulatory physical activity profiles of older adults. J Aging Phys Act.

[40] Yoo SH (2015). Predictors of senior center attendance in Korea: findings from a National Analysis. J Soc Serv Res.

[41] Whaley DE, Ebbeck V (1997). Older adults’ constraints to participation in structured exercise classes. J Aging Phys Act.

[42] Swan JH, Turner K, Shashidhara S, Sanders D (2013). Increased physical activity , physician recommendation , and senior center participation. Health (Irvine Calif).

[43] O’Neill C, Dogra S (2016). Different types of sedentary activities and their association with perceived health and wellness among middle-aged and older adults: a cross-sectional analysis. Am J Health Promot.

