# Supplementary material for: Physical activity benefits of attending a senior center depend largely on age and gender: a study using GPS and accelerometry data
Source: BMC Geriatr. 2020 Apr 15;20:134. doi: 10.1186/s12877-020-01527-6 (PMC7157997; doi:10.1186/s12877-020-01527-6)
Supplement: Supplementary file 1 — Additional file 1: Table S1 Multilevel linear regressions per type of daily physical activity and participant characteristics. [file 12877_2020_1527_MOESM1_ESM.docx]

| **Supplementary Table 1: Multilevel linear regressions per type of daily physical activity and participant characteristics.** | | | | | | | | | | | |
| --- | --- | --- | --- | --- | --- | --- | --- | --- | --- | --- | --- |
|  | **Sedentary Minutes^a^** | | |  | **Light PA minutes^b^** | | |  | **MVPA minutes^c^** | | |
|  | Coef | P>z | 95% Conf. Interval |  | Coef | P>z | 95% Conf. Interval |  | Coef | P>z | 95% Conf. Interval |
| **Senior Center** |  |  |  |  |  |  |  |  |  |  |  |
| No visit | Ref | - | - |  | Ref |  |  |  | Ref |  |  |
| Visit | -9.58 | <0.001* | -18.5; -0.6 |  | 8.77 | 0.04* | 0.0; 17.6 |  | 0.58 | 0.147 | -0.21; 1.37 |
| **Gender** |  |  |  |  |  |  |  |  |  |  |  |
| Women | Ref | - | - |  | Ref |  |  |  | Ref |  |  |
| Men | 34.10 | <0.001* | 5.7; 62.5 |  | -33.33 | 0.02* | -61.3; -5.3 |  | -0.76 | 0.223 | -1.98; 0.46 |
| **Age** |  |  |  |  |  |  |  |  |  |  |  |
| <75 y.o. | Ref | - | - |  | Ref |  |  |  | Ref |  |  |
| >= 75 y.o. | 19.06 | 0.204 | -10.3; 48.5 |  | -17.96 | 0.224 | -46.9; 11.0 |  | -1.09 | 0.089 | -2.35; 0.17 |
| **Device Weartime** | 0.83 | <0.001* | 0.8; 0.9 |  | 0.17 | <0.001* | 0.1; 0.2 |  | 0.00 | 0.041* | 0.00; 0.00 |
| **Health** |  |  |  |  |  |  |  |  |  |  |  |
| Good | Ref | - | - |  | Ref |  |  |  | Ref |  |  |
| Regular | 36.12 | 0.023* | 5.0; 67.3 |  | -34.24 | 0.029* | -64.9; -3.6 |  | -1.89 | 0.005* | -3.22; -0.56 |
| Poor | 60.32 | 0.098 | -11.1; 131.8 |  | -58.70 | 0.102 | -129.1; 11.7 |  | -1.68 | 0.281 | -4.73; 1.37 |
| **Rain** |  |  |  |  |  |  |  |  |  |  |  |
| No | Ref | - | - |  | Ref |  |  |  | Ref |  |  |
| Yes | 10.92 | 0.032* | 1.0; 20.9 |  | -10.99 | 0.028* | -20.8; -1.2 |  | -0.27 | 0.542 | -1.13; 0.60 |
| **Travel time to SC^d^** |  |  |  |  |  |  |  |  |  |  |  |
| <10 min | Ref | - | - |  | Ref |  |  |  | Ref |  |  |
| 10-20 min | -18.84 | 0.298 | -54.3; 16.6 |  | 19.19 | 0.282 | -15.8; 54.1 |  | -0.35 | 0.651 | -1.87; 1.17 |
| 11-20 min | -24.47 | 0.213 | -63.0; 14.0 |  | 23.85 | 0.218 | -14.1; 61.8 |  | 0.69 | 0.417 | -0.97; 2.35 |
| **Gender#Senior Center** |  |  |  |  |  |  |  |  |  |  |  |
| Women – No visit | Ref | - | - |  | Ref |  |  |  | Ref |  |  |
| Women – Visit | -18.20 | 0.003* | -30.3; 6.1 |  | 16.05 | 0.008* | 4.1; 28.0 |  | 1.94 | <0.001* | 0.88; 3.01 |
| Men – No visit | 26.96 | 0.071 | -2.3; 56.2 |  | -27.31 | 0.063 | -56.1; 1.5 |  | 0.35 | 0.609 | -1.00; 1.70 |
| Men – Visit | 27.62 | 0.069 | -2.2; 57.4 |  | -27.18 | 0.069 | -56.5; 2.2 |  | -0.65 | 0.377 | -2.09; 0.79 |
| **Age#Senior Center** |  |  |  |  |  |  |  |  |  |  |  |
| <75 y.o – No visit | Ref | - | - |  | Ref |  |  |  | Ref |  |  |
| <75 y.o – Visit | -17.94 | 0.019* | -32.9; -2.9 |  | 16.47 | 0.029* | 1.7; 31.2 |  | 1.32 | 0.052 | -0.01; 2.64 |
| >= 75 y.o. – No visit | 14.19 | 0.357 | -16.0; 44.4 |  | -13.48 | 0.375 | -43.2; 16.3 |  | -0.67 | 0.35 | -2.06; 0.73 |
| >= 75 y.o. – Visit | 9.22 | 0.556 | -21.5; 39.9 |  | -8.94 | 0.562 | -39.2; 21.3 |  | -0.48 | 0.521 | -1.96; 0.99 |
| **Age#Gender#Senior Center** |  |  |  |  |  |  |  |  |  |  |  |
| <75 y.o – Women – No visit | Ref | - | - |  | Ref |  |  |  | Ref |  |  |
| <75 y.o – Women – Visit | -15.23 | 0.128 | -34.9; 4.4 |  | 12.40 | 0.208 | -6.9; 31.7 |  | 2.73 | 0.002* | 0.98; 4.47 |
| <75 y.o – Men – No visit | 47.02 | 0.061 | -2.1; 96.2 |  | -45.69 | 0.065 | -94.2; 2.8 |  | -1.33 | 0.243 | -3.56; 0.90 |
| <75 y.o – Men – Visit | 25.11 | 0.326 | -25.0; 75.2 |  | -23.42 | 0.352 | -72.8; 25.9 |  | -1.77 | 0.146 | -4.15; 0.62 |
| >= 75 y.o. – Women – No visit | 27.64 | 0.178 | -12.6; 67.9 |  | -25.68 | 0.204 | -65.3; 14.0 |  | -1.88 | 0.041* | -3.69; -0.08 |
| >= 75 y.o. – Women – Visit | 7.88 | 0.706 | -33.1; 48.9 |  | -7.66 | 0.71 | -48.1; 32.8 |  | -0.42 | 0.671 | -2.36; 1.52 |
| >= 75 y.o. – Men – No visit | 44.17 | 0.041* | 1.9; 86.5 |  | -43.45 | 0.041* | -85.1; -1.8 |  | -0.63 | 0.513 | -2.53; 1.26 |
| >= 75 y.o. – Men – Visit | 55.74 | 0.011* | 12.9; 98.6 |  | -54.02 | 0.012* | -96.3;-11.8 |  | -1.93 | 0.058 | -3.93; 0.06 |
| **_cons** | -40.7 | 0.049* | -81.3; 0.2 |  | 38.85 | 0.056 | -1.1; 78.8 |  | 1.70 | 0.171 | -0.73; 4.14 |
| ^a^ Wald chi^2^(13) = 6539.65; Log likelihood = -3740.43; Prob > chi^2^ = 0.0000  ^b^ Wald chi^2^(10) = 293.19; Log likelihood = -3732.28; Prob > chi^2^ = 0.0000  ^c^ Wald chi^2^(10) = 36.95; Log likelihood = -2046.49; Prob > chi^2^ = 0.0001  ^d^ Self-reported walking travel time to the nearest senior center.  * *p* < 0.05 | | | | | | | | | | | |
